# Impact of Safety Message Generation Rules on the Awareness of Vulnerable Road Users

**DOI:** 10.3390/s21103375

**Published:** 2021-05-12

**Authors:** Tomás Lara, Alexis Yáñez, Sandra Céspedes, Abdelhakim Senhaji Hafid

**Affiliations:** 1 Department of Electrical Engineering, Universidad de Chile, Santiago 8370451, Chile; tomas.lara@ug.uchile.cl (T.L.); alexis.yanez@ing.uchile.cl (A.Y.); 2 School of Industrial Engineer, Universidad Santo Tomás, Santiago 8370003, Chile; 3 NIC Chile Research Labs, Universidad de Chile, Santiago 8370456, Chile; 4 Department of Computer Science and Operational Research, University of Montreal, Montreal, QC H3T IJ4, Canada; ahafid@iro.umontreal.ca

**Keywords:** awareness, cooperative ITS, message generation rules, vulnerable road user, vehicle-to-pedestrian communications

## Abstract

In the face of cooperative intelligent transportation systems (C-ITS) advancements, the inclusion of vulnerable road users (VRU), i.e., pedestrians, cyclists, and motorcyclists, has just recently become a part of the discussion. Including VRU in C-ITS presents new challenges, most notably the trade-off between the increase in VRU safety and the aggravation in channel congestion resulting from VRU-generated messages. However, previous studies mainly focus on network-related metrics without giving much consideration to VRU safety-related metrics. In this context, we evaluated such a trade-off with a study of motion-based message generation rules for VRU transmissions. The rules were analyzed using theoretical and simulation-based evaluations. In addition to studying the message generation rules using channel load metrics, such as channel busy ratio (CBR) and packet delivery ratio (PDR), we introduced a new metric: the VRU Awareness Probability (VAP). VAP uses the exchange of messages from active VRU to measure the probability of VRU detection by nearby vehicles. Results show that fixed message-filtering mechanisms reduce the overall channel load, but they could negatively impact VRU detection. We established the importance of quantifying the VRU awareness and its inclusion in C-ITS analysis because of its direct impact on VRU safety. We also discussed approaches that include VRU context and dynamism to improve the definition of message generation rules.

## 1. Introduction

Vehicular networking represents one of the critical elements to enable a diverse set of applications related to intelligent transportation systems (ITS). Such applications range from safety to convenience services bringing benefits to accident prevention, accident mitigation, traffic optimization, comfort, and many others [1].

In the safety area, several approaches have been proposed to detect (mobile) road users other than vehicles. The most common approach is passive detection, in which vehicles use different sensor technologies, such as video analysis and object detection based on non-visible light, to characterize the surroundings (e.g., signalization elements and presence of pedestrians) and to identify possible traffic threats involving other road users. Nevertheless, the need for line-of-sight (LoS) restricts the proper operation of the sensing devices in the passive approach. Moreover, because the LoS condition cannot be ensured in all situations, the passive approach is unsuitable for several safety use cases, as previously pointed out by many authors [2,3,4,5,6,7]. Consequently, cooperative communication-based approaches, called cooperative intelligent transportation systems (C-ITS), have emerged as a suitable alternative. In C-ITS, vehicles have sensing capabilities together with communication capabilities to actively broadcast information to other vehicles, other road users, and the infrastructure [8].

Despite the benefits that C-ITS brings to traffic safety, existing systems still fail to include all possible road users as active participants in the communication processes [8]. One relevant group that has not been fully considered is that of vulnerable road users (VRU), which includes a broad range of road users, such as pedestrians, cyclists, motorcyclists, and powered two-wheelers (PTWs) [6,9,10]. Road accidents are particularly harmful to this group of users. In fact, the World Health Organization (WHO) states that among the 1.35 million deaths associated with road traffic each year, half of these belonging to this group of road users. The lack of inclusion of these actors in the planning, design, and management of road operations explains a significant percentage of these fatalities [11].

### 1.1. Motivation

In recent years, several efforts have sought to reduce road traffic deaths and improve the safety of VRU using communication technologies. These efforts involve exchanging messages between vehicles and VRU using vehicle-to-pedestrian (V2P) communications; V2P includes the exchange of information between vehicles and any type of VRU. Different technologies are proposed for V2P, including Wi-Fi [12], cellular networks [13], dedicated short-range communication (DSRC) and Bluetooth [14], and radio frequency identification (RFID) tags [15].

In vehicular networks, VRU can take on active or passive roles. An active VRU participates in the communication process by sending information (e.g., location and speed), usually in a periodic manner, or following a particular set of transmission rules. A passive VRU waits for messages sent by vehicles and may reply only when it receives a message. Establishing the suitability of these roles for the effective integration of VRU in C-ITS is still an open challenge [6,16]. The authors in [16] report an active role may have better performance than a passive one in terms of safety-requirement fulfillment (e.g., time of detection before a collision). In addition, active VRU may help improving the perception that vehicles have about their environment. The active role of VRU may be deployed in parallel or as an additional feature to the development of cooperative perception messages (CPM) [17,18] in the search to improve the knowledge of different communication nodes in a vehicular environment. However, as the number of active VRU increases, network congestion could become severe and negatively impact the reception capabilities of all network nodes.

The congestion problem becomes even more critical when considering the radio access technology. For example, in the case of DSRC, there are concerns about the suitability of the technology to support the increase in network traffic associated with VRU. These concerns are mainly because this technology already suffers from network congestion on the Control Channel—used for the exchange of safety packets—when the network density increases [4,5,19]. The problem may become critical in some countries, like the US, if the push to reduce the spectrum allocation for DSRC continues [20].

Different proposals of decision systems address network congestion problems associated with the active participation of VRU. Typically, they manage the transmission rate or filter the transmissions of active VRU according to the context or motion characteristics. Such context-aware systems use environmental variables and VRU motion features (e.g., velocity) to modify the transmission rate of VRUs’ mobile devices. In general, the proposed message generation rules allow the overall reduction in network load measured by the channel occupancy [4,5,19].

Although the use of rules achieves a network load reduction, a crucial factor that has not been sufficiently evaluated is the ability of contextual-decision systems to maintain VRU awareness when applying message filtering. We define VRU awareness as the proportion of VRU detected by a vehicle over the total number of VRU nearby. We maintain that this parameter is critical in contextual-decision systems because of its direct relation to the capacity of vehicles to detect possible threats and avoid traffic accidents through the exchange of messages. Understanding the trade-off between reducing network congestion and maintaining an appropriate level of VRU awareness in contextual-decision systems is what motivated this work.

### 1.2. Contributions

In an effort to improve the safety of VRU, this work investigated the capacity of vehicles to identify nearby active VRU using contextual systems that apply message generation rules. The contributions of this paper can be summarized as follows:

- We defined a novel VRU awareness metric to analyze the impact of VRU message generation rules on the timely detection of VRU. Our proposed VRU awareness probability metric (VAP) measures the ability of a vehicle to detect the surrounding VRU as a function of the probability of successful message reception.
- We conducted a trade-off study based on channel busy ratio (CBR), packet delivery ratio (PDR), and VAP to analyze VRU message generation rules proposed in the literature [4]. For this purpose, we extend a vehicle-only IEEE 802.11p model to integrate VRU nodes in the vehicular communication network. In contrast to [4], our model does not separate the traffic of VRU and vehicles in different communication channels; instead, we considered a single shared channel scenario. The model also accounts for the effect of hidden terminals, caused by obstacles, to evaluate the impact of the network traffic generated by VRU.
- We developed a simulation tool to validate our theoretical model, which differs from previous works and tools that consider only pedestrians and cars (e.g., [4,5,19]). In this work, we also included bicycles and motorcycles, which present different mobility patterns compared to pedestrians. This inclusion is important because the mobility patterns directly affect the activation of the VRU message generation rules under study, especially those related to the position of VRU in urban scenarios.

The remainder of the paper is organized as follows. Section 2 reviews the related work, focusing on proposals of message generation rules; we also review the incorporation of awareness metrics in existing contributions. Section 3 describes the theoretical part of the methodology used in this study, presenting the message generation rules, the evaluation metrics, and the design of theoretical tests. Section 4 presents the simulation aspects used for the analysis of the message generation rules. Section 5 evaluates the impact of VRU message generation rules and discusses further improvements in the rules’ design. Finally, Section 6 concludes the paper and presents future work.

## 2. Related Work

Although contextual-based transmission mechanisms are a well-investigated field in vehicular communications, to date, similar techniques that focus on VRU do not present the same depth of exploration. Elements of vehicular context-based triggering systems were used as an important inspiration for this work. One example is the development of the context-based rules for collective perception messages (CPM) and the evaluation of the effectiveness of detection using such messages (closely related to awareness). CPM, standardized by the European Telecommunications Standards Institute (ETSI) [21], allow the exchange of sensor information among vehicles, increasing each vehicle’s environmental knowledge of the road [17].

The context-based rules include contextual variables of the detected objects, such as their movement, novelty, and speed changes to determine if a message is transmitted. Previous works have studied the channel load variations, derived from the transmissions triggered by these rules compared with a baseline case—a fixed message transmission rate. They also introduced metrics related to object awareness. In [17], the authors introduced two relevant metrics: the object awareness ratio (OWR) and the time between object updates. OWR measures the time between received CPM that contain information of the same object. In contrast, the time between object updates measures object-related awareness using the number of distinct acknowledged objects via CPM. Along this line of study, the authors in [18] measure the object awareness using the number of distinct acknowledged objects via CPM.

In the context of VRU communications, the authors in [19] developed a DSRC-based system for vehicle-to-pedestrian communications using smartphones. The system incorporates a context-awareness module to enable and disable the DSRC operation on the VRU side. First, the smartphone determines the pedestrian’s motion-state (i.e., stationary, walking, or running). The state information is used to turn off the message transmission and GPS signal reception in stationary pedestrians. The authors stated that the control mechanism allows the system to reduce power consumption and channel congestion, although the latter was not analyzed. They also discussed several challenges related to VRU inclusion in C-ITS, such as the spectrum and channel congestion. They proposed a mitigation alternative, termed *receive-only mode*, where mobiles are only allowed to receive. However, such a mode resembles a passive approach, which can reduce pedestrian detection compared to an active communication approach [16].

Sewalkar et al. [5] studied VRU transmissions through simulations. In the simulated scenarios, the authors studied two metrics, namely the channel busy percentage (CBP) and the beacon packet error ratio ($B_{p}ER$), to evaluate the impact of the inclusion of pedestrians to the existing vehicle-to-vehicle (V2V) communications. In light of the simulation results, the authors concluded that implementing critical safety applications in V2P communications is not possible using a fixed beaconing rate. To cope with this problem, the authors proposed a variable beaconing rate based on a context-aware system. The system, proposed by the authors, uses a context-sensitive clustering mechanism. This mechanism groups pedestrians and allows just one to send group information to vehicles, thereby reducing the number of exchanged messages.

Lee et al. [22] investigated communication channel overload along with battery-savings. They proposed a mechanism that considers clusters of VRU that exchange messages through WiFi Direct while a cluster leader exchanges messages with cars through IEEE 802.11. In terms of channel load, the system reduces the load by changing the state of the slave devices to sleep mode if the cluster leader detects a low velocity of surrounding vehicles. In contrast, when high vehicle velocity is detected, the devices transmit at a higher rate. A more straightforward approach is taken by the WiSafe system [12], which defers transmissions when pedestrians are detected to be indoors.

Bagheri et al. [23] proposed a system based on vehicle-to-cloud (V2C) and pedestrian-to-cloud (P2C) to exchange information between mobile nodes and the cloud, where the alerts are generated. The cloud sets the transmission rate of pedestrians lower or higher depending on the velocity of vehicles. If pedestrians are located indoors, the system stops the pedestrians’ message transmission, considering that they are in a safe place.

Rostami et al. [4] studied the problem of channel congestion in IEEE 802.11p. To reduce congestion, the authors proposed a context-based transmission system using three rules based on the motion and position of pedestrians. To test these rules, they carried out simulations and compared the decision system performance in contrast to a fixed rate mechanism based on CBP, packet error rate (PER), and a near-the-worst-case Inter Packet GAP (95% IPG). The results show an improvement of these metrics when applying the triggering rules. However, there was no evaluation of VRU detection capabilities. Based on the work in [4], in this paper, we studied both the system capacity to cope with channel congestion—as in the original paper—but also the effectiveness of the VRU detection. Furthermore, in contrast to [4], where they assumed a dedicated channel for pedestrians, we considered a shared channel among vehicles and VRU.

## 3. System Model

**Figure 1:**
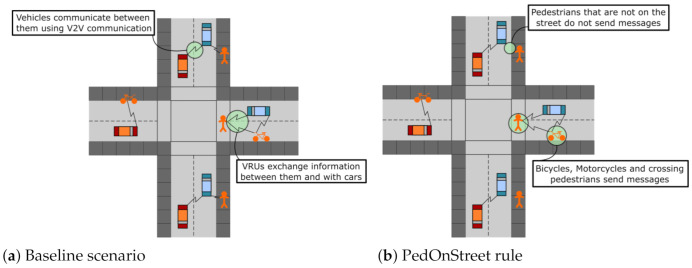
Rules representation.

The study scenario includes nodes of both types: VRU and cars equipped with IEEE 802.11p connectivity [24]. In addition to vehicle traffic, VRU also exchange safety messages with cars and other VRU to raise the context cooperative awareness. Therefore, in this network, VRU are defined as active nodes capable of sending information to their entire neighborhood. A representation of the scenario is illustrated in Figure 1a, where both vehicles and VRU establish bidirectional communication links.

Although the assumption that pedestrians, cyclists, and motorcyclists may be equipped with a communication unit that supports the IEEE 802.11p is not yet a reality, there have been initiatives for implementing a DSRC protocol stack in smartphones [19], with no need for additional elements. Moreover, given that smartphones are the most common communication device for VRU [8,16], the access technology options are extended to the use of cellular infrastructure and the Mode 3 and Mode 4 introduced for Cellular-V2X (C-V2X) communication [25,26,27]. Beyond the wireless technology in use, in this paper, it is of interest to evaluate the use and the availability of a shared communication channel. Such an analysis may establish a precedent when considering other technologies, such as C-V2X, and other architectures, such as heterogeneous networks [28,29].

The sets ped (i.e., pedestrians) and cycle (i.e., bicycles and motorcycles) form the complete set of vulnerable road users, VRU, where |ped|=P and |cycle|=C. Hence, VRU=ped∪cycle and |VRU|=V=P+C. From the set of VRU, there is only a subset of members that are actively transmitting messages, since the message generation rules cause some VRU to not always transmit. The subset of transmitting pedestrians is $ped_{tx}\subseteq ped$, where $\;P_{tx}\leq P$, whereas the subset of transmitting cycles is $cycle_{tx}\subseteq cycle$, where $\;C_{tx}\leq C$. Hence, the subset of transmitting VRU is $VRU_{tx}=ped_{tx}\cup cycle_{tx}$, where $V_{tx}=P_{tx}+C_{tx}$. It is worth noting that the cardinalities $P_{tx}$ and $C_{tx}$ heavily depend on the message generation rule. The scenario includes a set of vehicles, denoted by the set $car=\{car_{1},...,car_{N_{car}}\}$, where $\left|car\right|=N_{car}$. All cars are transmitting cooperative awareness messages at a fixed rate.

The proposed system model presents the message generation rules that govern VRU transmissions, together with the 802.11p model used to derive the evaluation metrics of the system. The notations used in this section are summarized in Table 1.

### 3.1. VRU Message Generation Rules

The generation of safety messages by VRU follows each one of the three rules introduced in [4] (see below). Note that the rules apply to outdoor VRU only.

1. **Pedestrian on Street (PedOnStreet):** Pedestrians only transmit messages when they are on the street. Other types of VRU like bicycles and motorcycles—grouped under the name cycle—are assumed to always be on the street, so they always transmit messages under this rule. A representation of this rule behavior is shown in Figure 1b, in which pedestrians who are not on the street make no transmissions.
2. **Moving VRU (MovVRU):** VRU transmit only when they are moving (VRU’s velocity is different from zero); thus, stationary VRU do not transmit.
3. **Multiple Transmission Rates (MultiTx):** This rule modifies the transmission rate ($\lambda_{MTx}$) of messages according to whether VRU are moving or not. In the case of a stationary VRU, the transmission rate is set to 2 Hz, while in the case of an in-motion VRU, the transmission rate is set to 5 Hz. $\lambda_{MTx}$ is defined in Equation (1), where *v* is the VRU velocity:
  (1) $$\lambda_{MTx}=\left\{\begin{array}{ccc} 2\;\mathrm{Hz} & if & v=0\;[m/s]\\ 5\;\mathrm{Hz} & if & v>0\;[m/s] \end{array}\right.$$

To evaluate the performance of these three rules, we also defined a baseline. In the baseline scenario, all VRU periodically generate safety beacons. We considered fixed transmission rates of 1 Hz, 2 Hz, 5 Hz, and 10 Hz.

### 3.2. IEEE 802.11p Model

For the theoretical analysis of the message generation rules and calculation of metrics, we adapted the IEEE 802.11p model proposed by Hassan et al. [30]. The authors address the problem of modeling the performance of the IEEE 802.11p protocol on a cooperative collision avoidance (CCA) system. Here, we briefly describe the model assumptions, performance metrics derivations, and the adjustments made to comply with our study scenario. The main assumptions in the model are [30]: (1) vehicles are considered in a stationary state during the communication interval; (2) transmission and sensing ranges are equivalent (value *R*), and they are the same for all nodes; (3) data packets arriving for transmission at a node follow a Poisson distribution, with the same parameter λ for all nodes; (4) the collision probability of each station is constant; (5) channel conditions are ideal within the transmission and sensing range, *R*; and (6) a highway scenario is assumed.

Although we focus on an urban scenario, we justify using the model in [30] since the transmission range of nodes using 802.11p is considerably wider than the street lane; hence, the linear distribution of nodes can be assumed to satisfy the assumption (6). However, we made modifications to consider nodes other than cars in the network. The notations used in this section are summarized in Table 1.

To calculate the probability of a busy channel, $p_{b}$, the authors in [30] employed the busy time approach: the time the radio senses the medium as occupied, which is a function of the packet transmission rate and the packet collision probability $p_{dc}$. The original calculation of $p_{b}$ in [30] is:

(2) $$p_{b}=\left(N_{tr}-1\right)\lambda T\left(1-\frac{p_{dc}}{2}\right).$$

In this equation, λ represents the rate of the Poisson process associated with the packet generation, and *T* is the packet transmission time, including the wait for a distributed coordination function (DCF) interframe space, DIFS. $p_{dc}$ is the packet collision probability when hidden nodes are not considered. As shown in Equation (2), there is a unique value for λ. Hence, we modified the model to consider multiple types of nodes—including VRU—in the network. Equation (3) is the new calculation of busy time, whereas Equation (4) is the new computation of $p_{b}$, which we now call CBR. To compute the cardinality of the sets $ped_{tx}$ (*P*), $cycle_{tx}$ (*C*), and car ($N_{car}$) we use the expression $Cardinality_{j}=1+2R\beta_{j}$, where $\beta_{j}$ is the density of the j-th category of transmitting nodes. For example, the number of transmitting pedestrians is computed as $ped_{tx}=1+2R\beta_{ped}$:

(3) $$BusyTime=T*(N_{car}\lambda_{car}+P\lambda_{ped}+C\lambda_{cycle}),$$

(4) $$CBR=BusyTime*(1-\frac{p_{dc}}{2}).$$

Here, we present the equations used for the computation of the collision probability and the probability of a node transmitting in an arbitrary time slot. As we consider arbitrary tagged nodes in each category of road users, the collision probability of the jth node category can be expressed as follows:

(5) $$p_{dc_{j}}=\left(1-\left(1-\rho_{j}\right)\left(1-p_{b}\right)\right)P\left(tx\right).$$

The probability of any transmission in an arbitrary time slot, defined as P(tx), can be defined as follows:

(6) $$P\left(Tx\right)=1-\left({\left(1-\tau \rho_{car}\right)}^{N_{car}-1}{\left(1-\tau \rho_{ped}\right)}^{P_{tx}-1}{\left(1-\tau \rho_{cycle}\right)}^{C_{tx}-1}\right),$$

where τ is computed as $\tau =\frac{1}{\bar{W}+1}$, with $\bar{W}$ being the mean value of the contention window. The packet collision probability, when considering hidden terminals ($p_{c}$), can be defined as follows:

(7) $$p_{c}=1-\left(1-p_{dc}\right)P\left(H_{1}\right)P\left(H_{2}\right),$$

where $P(H_{1})$ and $P(H_{2})$ represent the occurrence probability of two events in a hidden terminal scenario. $H_{1}$ is the event of a tagged vehicle transmitting, and none of the hidden terminals are transmitting. On the other hand, $H_{2}$ is when none of the hidden terminals start to transmit until the tagged vehicle finishes its transmission. Both $p_{dc}$ and $p_{c}$ only account for collisions between two packets.

### 3.3. Evaluation Metrics

To evaluate the generation rules, we propose a novel awareness metric, the VRU awareness probability (VAP), to account for the knowledge that vehicles have about VRU presence in their surroundings. Such a metric is complemented with the channel busy ratio (CBR) and the packet delivery ratio (PDR) to account for the channel load and successful delivery of packets, respectively.

To calculate CBR, we employ Equation (4). In the PDR case, we calculate the ratio between the successfully delivered packets and the total number of sent packets. To compute this ratio, we consider that the correctly delivered packets are the packets that do not present collisions. Considering this definition, PDR can be calculated using $p_{c}$ in Equation (7) for the case with hidden nodes. More specifically, PDR is computed as follows:

(8) $$PDR=1-p_{c}.$$

**Figure 2:**
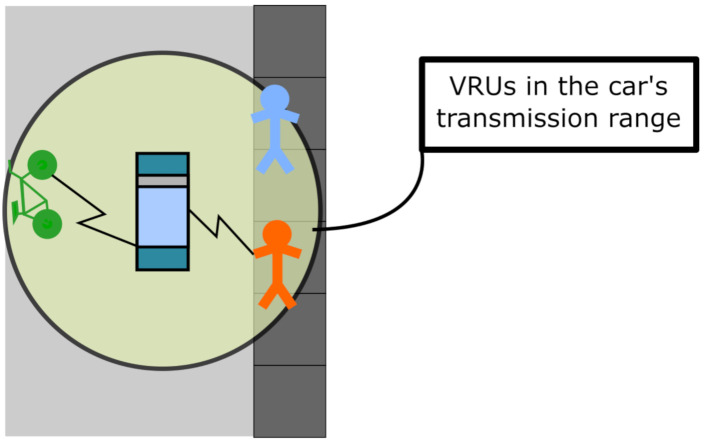
Representation of the interest zone around a tagged vehicle for VAP.

VAP assumes that VRU are only detected through the exchange of messages. Under this definition, the metric represents the proportion of correctly received VRU messages over the total number of VRU nodes in a tagged vehicle’s transmission range (Figure 2). VAP is calculated as a function of the transmission range of vehicles, the overall value of PDR, and the VRU density in the vehicle’s transmission range.

PDR was defined as the probability of the successful delivery of messages from a tagged node. For VAP computation, we considered that PDR is the same for every node; hence, the probability of the correct reception of VRU messages at the tagged vehicle is equal to the probability of effective delivery as seen by each VRU node that surrounds the tagged vehicle under study.

VAP can be expressed as follows:

(9) $$VAP=\frac{\sum_{i}P{\left(CR\right)}_{i}}{V};\;\;i\in VRU_{tx},$$

where $P{\left(CR\right)}_{i}$ is the correct car reception probability for each active VRU (denoted by *i*). To calculate $P{\left(CR\right)}_{i}$ we use the following expression:

(10) $$P{\left(CR\right)}_{i}=1-{\left(1-PDR\right)}^{Z_{i}};\;where\;Z_{i}\propto \lambda_{i},$$

where $Z_{i}$ represents the exponent of the loss rate computed as (1−PDR). $Z_{i}$ is proportional and not equal to the transmission rate $\lambda_{i}$ because, as shown in [31], the augmentation of the transmission rate, above a practical limit, does not contribute to the awareness capabilities if a time window of one second is considered. Thus, a transmission rate above approximately 2 Hz does not present considerable awareness improvements in neighborhood knowledge; therefore, a maximum value of Z=3 is used in this paper.

## 4. Simulation Analysis

To support the inclusion of active VRU in the wireless communication network, we developed an extension to the Veins framework [32] inspired by [33]; this extension allows the exchange of information between cars and VRU through the use of the IEEE 802.11p technology. Additionally, we can manage all protocol stacks for pedestrians and other kinds of VRU.

**Figure 3:**
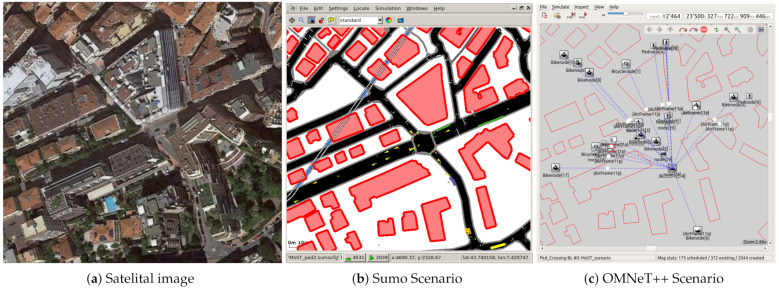
Views of the urban scenario with VRU.

We focused on evaluating an urban intersection scenario in which VRU naturally co-exist and interact with vehicular traffic. In particular, the buildings and roads were extracted from the simulation presented in [34], and VRU were included in SUMO using information from the road infrastructure presented in the micro-state of Monaco [35]. Figure 3 shows a satellite capture of the simulated intersection, together with captures from SUMO and OMNeT++.

**Figure 4:**
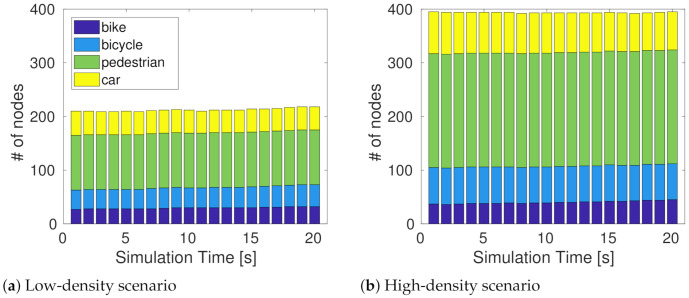
Composition of nodes in each scenario.

We extracted a portion of the map in [34], corresponding to a square of 200 m × 200 m and with a total road length of 1.14 km. Using car and VRU flows, we created scenarios with two different total densities (expressed in node per km^2^). In both scenarios, the number of pedestrians is considerably larger than the other nodes to better observe the impact of the message generation rules related to moving/stationary pedestrians. The two scenarios used for simulations are presented in Table 2. The values presented in Table 2 are mean values of the density because there are minor variations of the simulator’s node density in the analyzed time window (see Figure 4a for the low-density scenario and Figure 4b for the high-density scenario).

For the definition of the scenarios, we used as boundaries the density values used by Rostami et al. [4]. We computed these densities as the number of considered vehicles and VRU over the area simulated in their work. Between these boundaries, we defined the two scenarios presented in Table 2.

To study the performance of the different evaluation metrics, we simulated the scenarios using four message transmission rates: 1 Hz, 2 Hz, 5 Hz, and 10 Hz. The first three transmission rates were previously tested by [4]. The rates are used when any of the tested rules are activated. Table 3 presents a summary of all the parameters employed in the simulations.

### Validation of the Theoretical Model

We performed a comparative analysis of the theoretical model introduced in Section 3 and the simulation of a highly realistic intersection scenario. The parameters employed in the numerical results, for the different scenarios and rules, are listed in Table 4. Note that the parameters shown in Table 4 are consistent with the parameters shown in Table 3.

We evaluated the theoretical model using Matlab. In the model, we assumed a scenario geometry that approximates an intersection in a square grid of 200 m by 200 m to make it consistent with the simulations. It also considered the transmission power and sensibility threshold of the simulations, according to the values presented in Table 3. To account for the effect of obstacles at the intersection (e.g., buildings), which affect the hidden-node phenomena, we calculated the power decay using the path loss models WINNER+ [39]. Such models are commonly used in vehicular communications in urban areas. In the theoretical scenario, we used the formulation of the B1 case (“typical urban micro-cell”) corresponding to the non-line-of-sight case.

**Figure 5:**
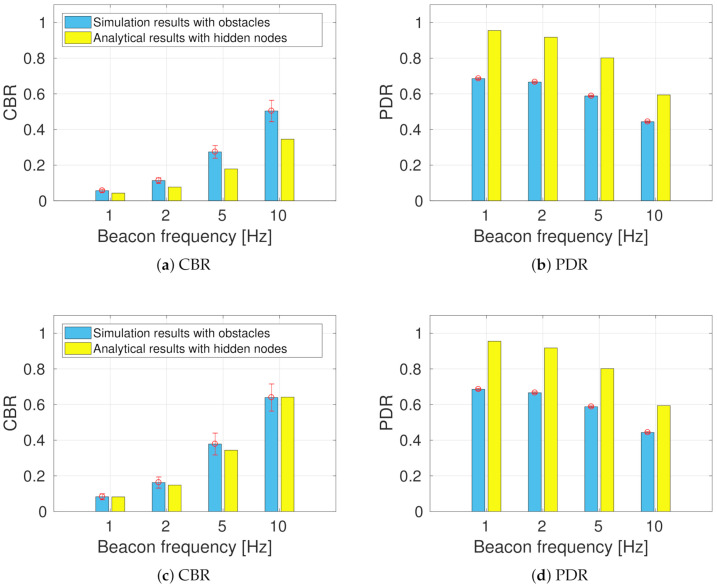
Comparison of theoretical model and simulations of a realistic intersection for different scenarios: low-density (**a**,**b**) and high-density scenarios (**c**,**d**).

After obtaining both numerical and simulation results, we compared the results of CBR and PDR to validate the precision of the model against a realistic scenario such as the one employed in the simulations. The results for the comparison of CBR and PDR are presented in Figure 5 for the low- and high-density scenarios, respectively. We observed that the theoretical model is quite close but slightly optimistic when compared to metrics obtained via simulations. Nevertheless, the model provides a good approximation that allowed us to perform a sensitivity analysis to understand the effects of the message generation rules in all the performance metrics: VAP, CBR, and PDR.

## 5. Evaluating the Impact of VRU Message Generation Rules

This section evaluates the impact of the message generation rules that control the sending of VRU context-awareness messages. First, we obtain the baseline results, where VRU send messages at a fixed rate (i.e., no message generation rules are in place). We then analyze the metrics considering the use of the message generation rules. In both analysis (i.e., with and without generation rules), we consider the same two scenarios and density compositions as shown in Table 2 and evaluate the impact via the theoretical model and the realistic simulations.

In the following step, we study the variations of the node behavior. Hence, we vary the proportions of moving pedestrians and cycles, as well as the percentage of pedestrians on the street. Such a sensitivity analysis is only performed with the theoretical model, given the limitations of modifying the proportions of nodes at will in the simulation tool because it uses realistic traces [32].

### 5.1. Analysis of Baseline VRU Transmissions

**Figure 6:**
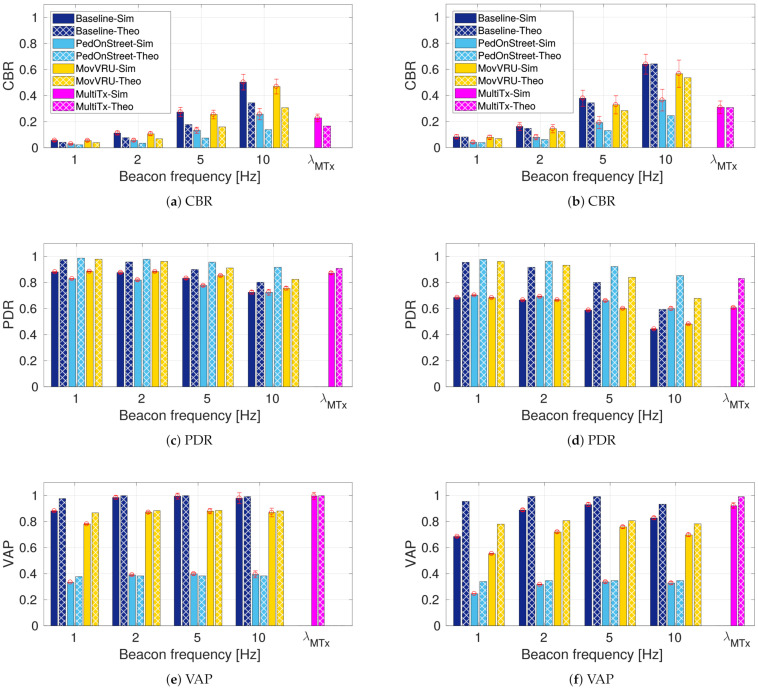
Simulation results for the low-density (**a**,**c**,**e**) and high-density scenarios (**b**,**d**,**f**).

First, we analyze the performance of the baseline rule in the two scenarios defined in Table 2. The baseline rule results in terms of CBR, PDR, and VAP are presented as the blue bars in Figure 6. In general terms, we observe that all metrics present better performance (i.e., lower CBR, higher PDR, and higher VAP) when evaluated in the low-density scenario (Figure 6a,c,e) as compared to the high-density scenario (Figure 6b,d,f). Such behavior is expected, given that the three metrics are dependent on the overall number of exchanged messages.

Our proposed awareness metric (VAP) provides additional, useful information when the frequency is varied. In the low-density scenario (Figure 6e), we observe that the awareness capabilities offer good performance with VAP values close to 1.0 in both theoretical and simulated results. The difference between beaconing rates is only significant when passing from 1 Hz to 2 Hz, showing an increase of about 1% in the theoretical model and 10% in the simulation results. The high-density scenario (Figure 6f) gives us more insights into the VAP behavior. Here, the simulation results exhibit a more considerable difference when varying the beaconing frequency of VRU. Following the Baseline-Sim bar, we observe that there is an increase in VAP when the beacon frequency varies from 1 Hz to 5 Hz. When the frequency is increased from 5 Hz to 10 Hz, we observe that the VAP value decreases by nearly 15%.

VAP variations can be explained by observing the formulation in Equation (9). VAP has a non-linear relation with PDR. In Equation (9), the exponent stays the same in the 5 Hz and 10 Hz case, while PDR decreases due to the increase in the number of packet collisions (see Figure 6d). The decrease in PDR is higher in the high-density case; hence, VAP reaches a breaking point where an increase in beaconing rate tends to lower the awareness rather than increase it. Figure 6f shows that this break occurs at a beaconing frequency of 5 Hz.

### 5.2. Comparison of Baseline and Message Generation Rules

Here, we present and discuss the results obtained for CBR, PDR, and VAP for the low and high-density scenarios when the message generation rules are in place. The x axis presents the data rates used by VRU to transmit when using each rule. When the MultiTx rule is in place, there is no dependency on the frequency; hence, it is labeled as $\lambda_{MTx}$—as described in (1).

In terms of channel load, Figure 6a,b show the CBR variations as a function of the rules, beaconing frequency, and scenario density. We observe that the most considerable difference is observed between the baseline rule and the PedOnStreet rule. For example, we observe that in the low-density scenario (Figure 6b), CBR decreases by 20% for the 5 Hz beaconing rate and by 30% for the 10 Hz beaconing rate. In addition, as expected, the higher-density scenario (Figure 6b) exhibits a higher CBR compared to the low-density scenario (Figure 6a). The CBR’s decrease when applying the PedOnStret rule is explained by the fact that most pedestrians are on the sidewalk, and hence not transmitting according to the PedOnStreet rule. The MultiTx rule exhibits a difference that changes from nearly 10% when compared to the 5 Hz baseline to 35% when compared to the 10 Hz baseline. The MovVRU rule has a similar behavior as the MultiTx rule since both depend on the VRU movement characteristics. The smaller decrease when using these rules is also explained by the scenario composition, where approximately 90% of VRU are moving.

The other metric that was used to quantify channel load was PDR. We present PDR results in Figure 6c,d. For both scenarios, this metric exhibits a relatively constant level for the beacon frequencies of 1 Hz, 2 Hz, and 5 Hz. In the 10 Hz frequency, the value of PDR decreases by approximately 15% with respect to its value in the 5 Hz frequency for the high-density scenario (Figure 6d). In terms of rules, analyzing the 10 Hz beaconing rate in the high-density scenario, we observe an increasing tendency for PDR. When comparing the baseline and the PedOnStreet rule, we observe an increase of about 20% in the high-density scenario. The improvements in these metrics are also observed when using the MovVRU and MultiTx rules with the rest of the tested rates.

Despite the improvements observed in terms of channel load, VRU awareness demonstrates a different behavior. VAP results are presented in Figure 6e,f for the low-density and high-density scenarios. In the following, we analyze the high-density scenario with a beaconing rate of 10 Hz. The most critical case in terms of VAP corresponds to the one when the PedOnStreet rule is used. When we compare the simulated baseline with the PedOnStreet rule, we observe a reduction of about 50% in VAP; this indicates that an additional 50% of pedestrians is not detected through cooperative communications. When applying the PedOnStreet rule in this scenario, only a few pedestrians, the ones that are actually on the street, are allowed to send messages; all other pedestrians (98%) are not detected.

The VAP decrease is less critical under the MovVRU rule, showing a variation of about 10% when compared to the simulated baseline. A higher number of moving VRU explains this smaller decay. The case of the MultiTx rule is interesting in terms of awareness analysis. Figure 6f shows that, under the MultiTx rule, VAP increases by about 10% compared to the baseline, reaching a VAP value near a 95%. The behavior can be explained by the formulation of this rule and the improvements exhibited in CBR and PDR. While the MultiTx causes a PDR increase, instead of deferring the VRU transmissions, it changes their transmission rate. The fact that VRU do not stop their communications but change the beaconing rate means that every node has the possibility of being detected, but each node’s message delivery probability is different.

We conclude that message generation rules can have different and opposite effects on channel load and VRU awareness. This is shown most notably with the PedOnStreet rule, where the most significant decrease in the channel load came with the most considerable decrease in VAP compared to the other rules.

### 5.3. Node Behavior Variation

Using the theoretical model (see Section 3), we perform a sensibility analysis, varying the percentage of active nodes when applying each rule. The percentage of active VRU is determined by the applied rule and the scenario characterization (proportion of moving VRU and ratio of pedestrians on the street). For this analysis, we consider a transmission rate of 5 Hz for VRU. We study the difference (in percentage) between the values of the selected metrics—CBR, PDR, and VAP—when the different rules are applied and when the baseline case is considered. We study both the case of the low-density and high-density scenarios.

**Figure 7:**
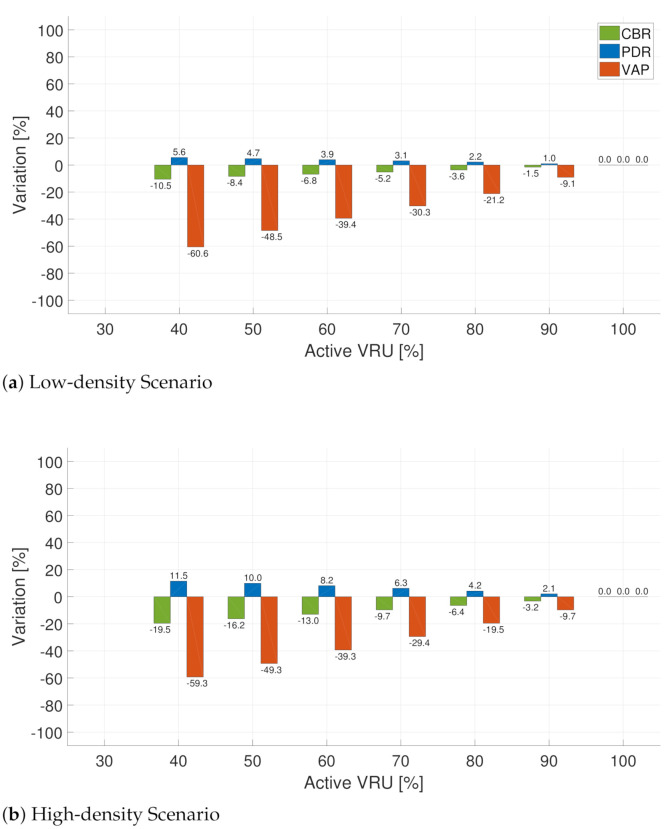
Difference(percentage) of CBR, PDR, VAP between the use of the baseline and PedOnStreet rule in two scenarios: low-density (**a**) and high-density scenarios (**b**).

First, we present the results when applying the PedOnStreet rule. In this case, the moving variable is the percentage of pedestrians on the street; cycles are assumed to be always on the street. We vary the percentage of pedestrians on the street between 0% and 100%. Figure 7 shows the results for the low-density (Figure 7a) and high-density (Figure 7b) scenarios. The graphs start at an activation value different from zero because of the presence of bicycles and motorcycles—always active under this rule—as shown in Table 2.

From Figure 7, we observe that the pedestrians’ behavior heavily determines the metrics variations. This effect is more evident in the VRU awareness capabilities reflected by VAP. If we focus on the range of active VRU between 40% and 70%, we observe different behaviors for each metric. For the low-density scenario (Figure 7a), in the case of CBR, we observe a decrease of 10.5% and 5.2% when the percentages of active VRU are 40% and 70%, respectively. Analyzing the same metric in the high-density scenario (Figure 7b), we observe a decrease when analyzing the same activation percentages. Figure 7 shows that PDR variations under the same conditions show a similar tendency as CBR, with the most relevant improvements (increases) at lower activation percentages.

**Figure 8:**
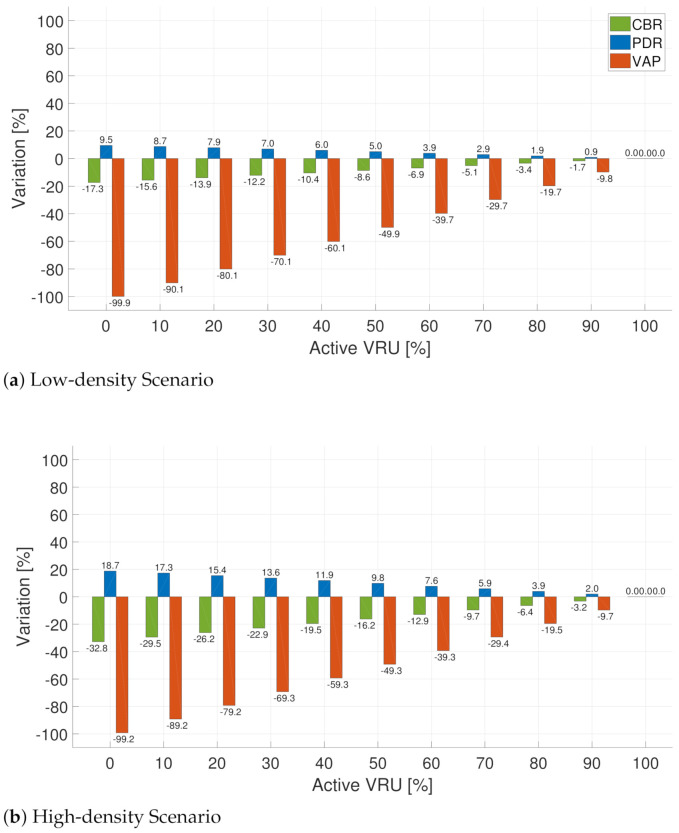
Difference (percentage) of CBR, PDR, VAP between the use of the Baseline and MovVRU rule in two scenarios: low-density (**a**) and high-density scenarios (**b**).

Despite the improvements in PDR and CBR, VAP decreases. For example, in the high-density scenario (Figure 7b), while the CBR values improve, the effect of the rule on VAP is a reduction of 60.6% and 30.3% when the percentages of active VRU are 40% and 70%, respectively. Results for the MovVRU rule (Figure 8) present the same tendency as that obtained for the PedOnStreet rule, showing an improvement of the channel load metrics while negatively affecting the VRU awareness capabilities. When using the MovVRU rule, the negative effect on VAP could be more severe because even lower percentages of VRU activation may occur. We consider this behavior critical when formulating message generation rules because improvements in terms of channel load—a reduction in CBR and an increase in PDR—could be accompanied by harmful effects on VAP due to the transmission deferral associated with the application of rules.

**Figure 9:**
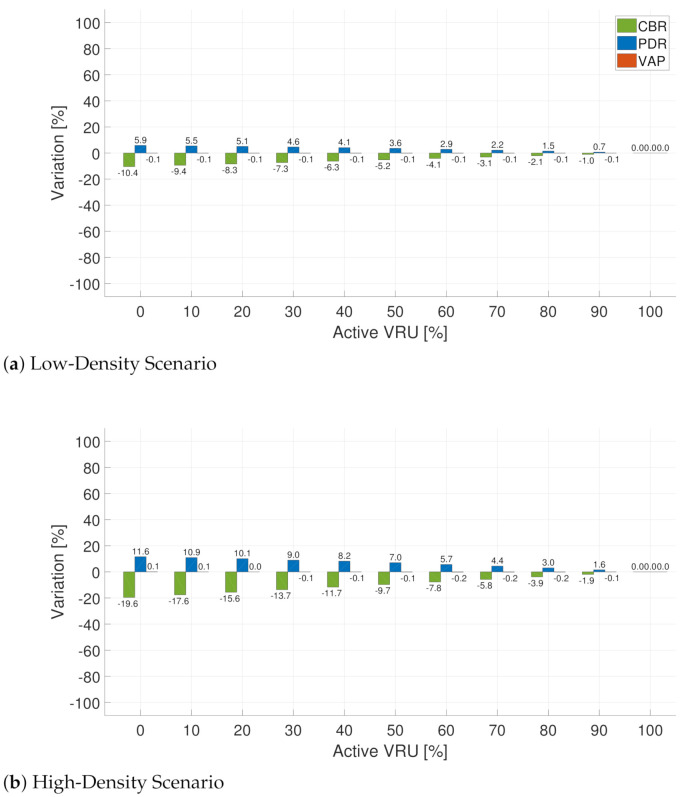
Difference (percentage) of CBR, PDR, VAP between the use of the baseline and MultiTx rule in two scenarios: low-density (**a**) and high-density scenario (**b**).

In contrast with the previous rules, the evaluatio of the MultiTx rule (Figure 9) differs in its behavior when the node activation percentage varies. From the congested scenario (Figure 9b), we observe that CBR decreases in the low activation portion while PDR increases. A remarkable difference when using this rule is the behavior of VAP. While in previously tested rules, this metric is severally affected in low-activation scenarios, when using the MultiTx rule, we observe that VAP presents a slight variation compared to the baseline; this means the VAP value is near 1.0, according to Figure 6f. This can be explained by the fact that even when VRU are stationary, they still send messages but at a lower rate. This means that the set of non-active VRU is not directly discarded from being detected by vehicles.

We chose DSRC as the access technology for the current study mainly because of its extended use and research. Other access technologies of interest, such as C-V2X, may vary on their levels of PDR, CBR, and VAP; however, we believe that our proposed analysis can apply to C-V2X technology with minor modifications to the metrics’ measurements. Despite the difference in the level of congestion and the awareness probability between the two technologies, the results, particularly in PDR and VAP, should follow the same trend [40,41]. The authors in [40] show that PDR, when using C-V2X, exhibits a slighter decrease compared to DSRC when the distance between the transmitter and the receiver varies; however, the decreasing trend is similar for both technologies. The authors in [41] compare the performance of IEEE 802.11p and LTE-V2X (precursor of C-V2X) using freeway and urban scenarios. The comparison results show that PRR (packet reception ratio), a metric related to PDR, follows a similar trend but with a smaller decrease in the LTE-V2X case for urban scenarios.

### 5.4. Variation for MultiTx Rule

Among the message generation rules under study, the MultiTx is the rule that shows the best performance in terms of the trade-off between congestion—measured through CBR and PDR—and awareness (VAP). The MultiTx rule varies VRU transmission frequency between 2 Hz and 5 Hz when the VRU are stopped or in movement, respectively (see Section 3.1). These arbitrary values were defined by the original formulation of the rules [4]. In this section, we perform a sensibility analysis, modifying the frequency pairs of the MultiTx rule, to investigate the effects of these variations on the performance metrics. We test four new combinations of frequencies (see Table 5).

**Figure 10:**
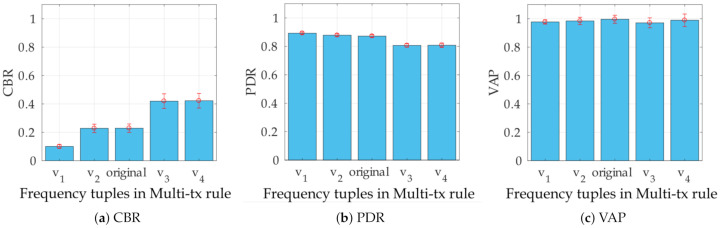

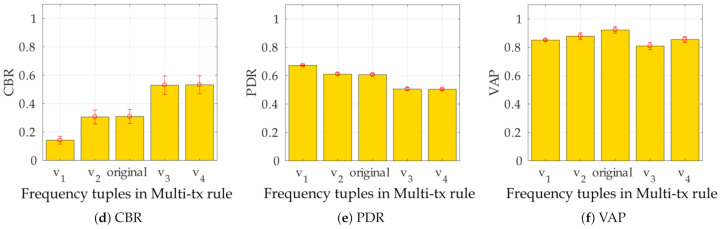
Simulation results for the variations from MultiTx rule (denoted by $v_{1},v_{2},v_{3},v_{4}$ and original in *X* axis); first row corresponds to low-density scenario (**a**–**c**) and second row corresponds to high-density scenario (**d**–**f**).

Figure 10a–c show the results for CBR, PDR, and VAP for the low-density scenario while Figure 10d–f show the results for CBR, PDR, and VAP for the high-density scenario. In both low-density and high-density scenarios, the behavior of PDR and CBR worsens when the beaconing frequencies increase. For example, in Figure 10a,b, CBR increases by 32.3% by using combination $v_{4}$ instead of $v_{1}$, whereas PDR decreases by 8.5%. Similarly, in Figure 10d,e, CBR increases by 39.1% by using combination $v_{4}$ instead of $v_{1}$, whereas PDR decreases by 16.8%.

Although the new combination of frequencies have an impact on PDR and CBR, VAP appears almost insensitive to the frequencies variations in the low-density scenario (see Figure 10c). In the high-density scenario, Figure 10f shows that the original frequencies (i.e., 5 Hz and 2 Hz) result in a better VAP. In this scenario, we observe a counterintuitive behavior of VAP for combinations $v_{3}$ and $v_{4}$: although PDR decreases (see Figure 10e), VAP shows a decrease for the same combinations, with respect to the original frequencies. Such a behavior can be explained by analyzing Equation (9). Figure 10e shows that PDR is lower in $v_{4}$, compared to $v_{3}$, which should negatively impact VAP. However, if there is an increase in the beaconing frequency, the number of opportunities for VRU to be detected increases. These two factors produce the difference in the trend followed by VAP in Figure 10.

### 5.5. Summary of Results

This section presented a study of a set of message generation rules based on channel load (CBR and PDR) and VRU awareness capabilities (VAP). As demonstrated in Section 5.1, the natural behavior of channel load is to grow when the traffic and VRU increase. This section also validated our theoretical adaptation of the model defined in [30] taking the simulations as reference. In Section 5.2, we discussed the effects of the various message generation rules on two predefined scenarios. The results showed that the more restricting rules in terms of transmission deferral—PedOnStreet and MovVRU—tend to reduce the channel load more significantly than the MultiTx rule. However, these improvements come with a reduction in VAP due to the more significant percentage of VRU not sending messages to their neighbors. The MultiTx rule proves to be the most suitable in the tested scenarios. Finally, in Section 5.3, our sensibility analysis showed the benefits and drawbacks that message generation rules could create in different mobility scenarios. The results of this section overall support the notion of defining more dynamic and adaptive message generation rules that successfully function in a more diverse set of conditions.

In the following, we summarize our findings based on the results obtained studying the rules proposed in [4]. Simple rules can be efficient in computational costs; however, they do not consider several features that can help in the transmission decision making. These rules do not consider the VRU context in their formulation. The context can be used to define the danger levels for different VRU. Moreover, the strict formulation of these rules may be not applicable for dynamic scenarios; indeed, the urban mobility of cars and VRU may lead to an excessive transmission of messages or a reduction in the awareness due to over filtering mechanisms. Several existing contributions emphasize the importance of the VRU intention recognition for the safety of VRU and the prevention of accidents [42]; VRU intention can be also used to help in the filtering process of messages. In the following, we briefly describe the key existing contributions that could help to develop message generation rules while taking into account the VRU context, intention, and dynamism.

With respect to context, the most common feature is to defer transmission when pedestrians are detected to be indoors; indoor sleeping mode was already considered in [4] and the WiSafe system [12]. The authors in [23] expanded the indoor deferral and included the case when pedestrians are stopped. Using V2C (vehicle-to-cloud) and P2C (pedestrian-to-cloud) communications, the proposed system [23] also incorporates beaconing management based on the distance between cars and pedestrians. More specifically, vehicles and moving pedestrians send beacons periodically; however, when the system does not detect possible threats to pedestrians, it commands to reduce the beaconing frequency. When the system detects a possible threat, it alerts pedestrians and orders them to change their beaconing frequency to 10 Hz. The authors in [43] defer the message transmission if any of the following conditions are met: static VRU, VRU indoors, VRU on a vehicle, or VRU in parks or safe regions. There are also solutions that use clusters (e.g., [22]), where the VRU cluster head exchanges messages with neighboring vehicles and instructs the VRU cluster members to transmit messages only when the speed of vehicles is high.

With respect to the VRU intention, several contributions propose to use visual sensors (mainly cameras), thermal sensors, RADAR (radio detection and ranging) technology, and LiDAR (light detection and ranging) to determine the intention [42]; the focus was mainly on pedestrians crossing intention and bicycle turning prediction. The authors in [44] used convolutional neural networks (CNNs) to determine the intention of pedestrians to cross a street. They used the same methodology to determine a cyclist intention to make a turn, assuming that she follows traffic rules to indicate maneuvers (arm signals). The authors in [45] proposed detecting the intention of vehicles to improve the safety of cyclists against the right turns of vehicles. The authors in [46] proposed to determine pedestrian intention based on video, using a latent-dynamic conditional random field model, and images including a laser scanner; they use recurrent neural networks, specifically a long short-term memory network with attention mechanism. The authors in [47] proposed a hidden Markov model, based on the use of 3D positions and displacements of 11 joints located along the pedestrian body, to determine pedestrian intent.

With respect to dynamic rules, we consider that the dynamic nature of the vehicular environment requires context-aware and adaptive rules. This is the subject of our current research; more specifically, we are developing a decision system, based on machine learning techniques, that allows to dynamically select between DSRC and C-V2X (cellular–vehicle-to-everything) in a heterogeneous network.

## 6. Conclusions

One of the main obstacles to the correct deployment of VRU systems is channel congestion. Several message generation rules have been proposed to cope with this problem. However, despite the benefits of this kind of rules in channel congestion, the effect on VRU detection has not been previously studied. In this paper, we introduced a novel VRU awareness metric—VRU awareness probability (VAP)—and studied existing message generation rules on channel load and VRU awareness. We performed the study using realistic simulations in Veins and theoretical models implemented in Matlab. Using these tools, we performed an analysis with real mobility traces and a sensibility study based on the variation of rule activation. To the best of our knowledge, the study of message generation rules with a scope including VRU awareness capabilities is new in the context of VRU active communications.

From our analysis, we observed that despite the benefits that message generation rules can bring in terms of channel congestion relief, there may be adverse effects on VRU detection capabilities mainly due to the lack of transmission in the presence of specific motion characteristics. We also showed that the degradation in detecting VRU through communications could be even more critical in scenarios with large proportions of VRU that do not satisfy the transmission criteria of fixed rules.

Overall, we consider our main contribution is to call attention to the importance of including VRU awareness analysis when formulating message generation rules in C-ITS systems. We defined a new metric that can support this additional evaluation. Finally, we proposed research directions to meet the challenges of adding VRU signals to the communication mix. In the following, we present a list of these challenges:

- Explore different access technologies: The use of other access technologies is an open field of research. One example of these technologies is C-V2X, a technology developed by 3GPP specifically designed for V2X communications. Works on C-V2X have shown improvements compared to DSRC [41]; however, more evaluations and implementations remain an open field. Another factor that adds interest to this technology is that FCC recently allowed part of the DSRC-dedicated spectrum to be used by C-V2X [20]. Another technology that may get the community’s attention is the IEEE 802.11p update named IEEE 802.11bd [48,49].
- Multiple access technologies: The use of different access technologies in a heterogeneous architecture could also be considered for channel load reduction without necessarily reducing VRU awareness. Current access technologies, such as DSRC, WiFi, and C-V2X, could coexist in the vehicular environment. There is a number of contributions [17,18,29,50] that consider multiple access technologies; however, they mainly focus on vehicular communications without the inclusion of traffic from active VRU.
- Position-based rules: Another line of research is the exploration of message generation rules that combine the position and direction of VRU with the motion-based policies to filter unnecessary messages. For example, rules could stop the transmissions of pedestrians considered to be in a safe zone (at a certain distance from the street or in a safe space, such as parks [43]) or walking away from the street.
- Clustering: As indicated by ETSI [51], the clustering of VRU should be considered as an alternative to managing large numbers of VRU. In this approach, future research could explore the dissemination or transmission selection schemes based on groups instead of individual VRU.

## Figures and Tables

**Table 1:** Notation table.

Symbol	Description
ped	Set of pedestrians in a vehicle’s transmission range
cycle	Set of bicycles and motorcycles in a vehicle’s transmission range
VRU	Set of vulnerable road users in a vehicle’s transmission range
car	Set of vehicles
$ped_{tx}$	Set of transmitting pedestrians in a vehicle’s transmission range
$cycle_{tx}$	Set of transmitting bicycles and motorcycles in a vehicle’s transmission range
$VRU_{tx}$	Set of transmitting vulnerable road users in a vehicle’s transmission range
*P*	Cardinality of the pedestrian’s set
*C*	Cardinality of the cycles’ set
*V*	Cardinality of the VRU’s set
$N_{car}$	Cardinality of the car’s set
$P_{tx}$	Number of pedestrians sending messages
$C_{tx}$	Number of cycles sending messages
$V_{tx}$	Number of VRU sending messages
CBR	Channel busy ratio
PDR	Packet delivery ratio
VAP	VRU awareness probability
$P{\left(CR\right)}_{i}$	Correct car’s reception probability for a VRU transmission (*i*)
$Z_{i}$	Exponent of the loss rate
$\lambda_{i}$	Transmission rate.
$p_{b}$	Probability of sensing a busy channel
*T*	Total transmission time of a packet
$p_{dc}$	Packet collision probability when hidden nodes are not considered.
$\beta_{j}$	Linear density of the j-th node (node/km)
*R*	Node transmission range (km)
$\rho_{j}$	Queue utilization of the j-th node.
$p_{c}$	Collision probability considering hidden terminals
$P(H_{1})$	Probability that no hidden node is transmitting when a tagged node is transmitting
$P(H_{2})$	Probability that no hidden terminal starts transmitting until a tagged vehicle finishes transmission.

**Table 2:** Description of tested scenarios.

	Scenario	Low-Density	High-Density
**Density (node/km^2^)**	Cars	38	66
	Pedestrians	89	186
	Cycles	54	93
**Behavior (%)**	Moving pedestrians	94.44	75.63
	Moving cycles	78.90	91.68
	Pedestrians on street	0.86	2.10

**Table 3:** Simulation parameters.

Physical Layer	
Frequency	5.89 GHz [36]
SimplePathLoss model	α=3.0 [37]
Transmission power	300 mW [38]
Receptor Ssnsitivity	−100 dBm [38]
Thermal noise	−110 dBm [38]
Antenna type	Monopole [38]
**Link layer**	
Bit rate	6 Mbps [38]
Contention window	[15, 1023] [36]
Slot time	13 µs [36]
SIFS	32 µs [36]
DIFS	58 µs [36]
**Messages**	
Beaconing frequency	{1,2,5,10} [Hz]
Beacon size	200 bytes
**Vehicular traffic**	
Vehicular density	{150,279} [veh/km]
Vehicle types	Buses, cars
	pedestrians, bicycles
	and motorcycles
**Simulation**	
Simulation time	20 [s]

**Table 4:** System model parameters.

Parameter	Value
Maximum backoff window size (W)	16
Transmission range (R)	0.366 km
Slot size (σ)	13 µs
DIFS	58 µs
Data rate ($R_{d}$)	6 Mbps
Packet arrival rate (λ)	{1,2,5,10} packets/s
Packet length (airframe)	200 Bytes

**Table 5:** Variation of the MultiTx rule.

Variation	Beacon Frequency in Hz	
	**Moving VRU**	**Stopped VRU**
$v_{1}$	2	1
$v_{2}$	5	1
original	5	2
$v_{3}$	10	1
$v_{4}$	10	2

## Abbreviations

The following abbreviations are used in this manuscript:

LoS	Line of Sight
C-ITS	Cooperative Intelligent Transportation Systems
VRU	Vulnerable Road Users
V2P	Vehicle-to-Person
RFID	Radio Frequency Identification
DSRC	Dedicated Short-Range Communications
FCC	Federal Communications Commission
CBR	Channel Busy Ratio
VAP	VRU Awareness Probability
PDR	Packet Delivery Ratio
V2X	Vehicle-to-Everything
CPM	Collective Perception Message
ETSI	European Telecommunications Standards Institute
OWR	Object Awareness Ratio
CBP	Channel Busy Percentage
$B_{p}ER$	Beacon Packet Error Ratio
PER	Packet Error Rate
95% IPG	Near-the-Worst-Case Inter Packet GAP
NLOS	Non Line of Sight
CCA	Cooperative Collision Avoidance
DCF	Distributed Coordination Function
DIFS	DCF Interframe Space
RMSE	Root Mean Squared Error
V2C	Vehicle-to-Cloud
P2C	Pedestrian-to-cloud
LTE-V2X	Long-Term Evolution-Vehicular-to-Everything
RADAR	Radio Detection and Ranging
LiDAR	Light Detection and Ranging
CNN	Convolutional Neural Networks
C-V2X	Cellular–Vehicle-to-Everything
PRR	Packet Reception Ratio

## References

[1] Karagiannis G., Altintas O., Ekici E., Heijenk G., Jarupan B., Lin K., Weil T. (2011). Vehicular networking: A survey and tutorial on requirements, architectures, challenges, standards and solutions. IEEE Commun. Surv. Tutor..

[2] David K., Flach A. (2010). Car-2-x and pedestrian safety. IEEE VEhicular Technol. Mag..

[3] Anaya J.J., Merdrignac P., Shagdar O., Nashashibi F., Naranjo J.E. (2014). Vehicle to pedestrian communications for protection of vulnerable road users. Proceedings of the 2014 IEEE Intelligent Vehicles Symposium Proceedings.

[4] Rostami A., Cheng B., Lu H., Kenney J.B., Gruteser M. Performance and channel load evaluation for contextual pedestrian-to-vehicle transmissions. Proceedings of the First ACM International Workshop on Smart, Autonomous, and Connected Vehicular Systems and Services.

[5] Sewalkar P., Krug S., Seitz J. (2017). Towards 802.11 p-based vehicle-to-pedestrian communication for crash prevention systems. Proceedings of the 2017 9th International Congress on Ultra Modern Telecommunications and Control Systems and Workshops (ICUMT).

[6] Scholliers J., Van Sambeek M., Moerman K. (2017). Integration of vulnerable road users in cooperative ITS systems. Eur. Transp. Res. Rev..

[7] Casademont J., Calveras A., Quiñones D., Navarro M., Arribas J., Catalan-Cid M. (2019). Cooperative-Intelligent Transport Systems for Vulnerable Road Users Safety. Proceedings of the 2019 7th International Conference on Future Internet of Things and Cloud (FiCloud).

[8] Rufino J., Silva L., Fernandes B., Almeida J., Ferreira J. (2018). Empowering Vulnerable Road Users in C-ITS. Proceedings of the 2018 IEEE Globecom Workshops (GC Wkshps).

[9] Silla A., Rämä P., Leden L., Van Noort M., de Kruijff J., Bell D., Morris A., Hancox G., Scholliers J. (2017). Quantifying the effectiveness of ITS in improving safety of VRUs. IET Intell. Transp. Syst..

[10] ETSI (2019). Intelligent Transport Systems (ITS); Vulnerable Road Users (VRU) awareness; Part 1: Use Cases Definition; Release 2.

[11] World Health Organization (2018). Global Status Report on Road Safety 2018.

[12] Ho P.F., Chen J.C. (2016). Wisafe: Wi-fi pedestrian collision avoidance system. IEEE Trans. Veh. Technol..

[13] Zadeh R.B., Ghatee M., Eftekhari H.R. (2017). Three-phases smartphone-based warning system to protect vulnerable road users under fuzzy conditions. IEEE Trans. Intell. Transp. Syst..

[14] Anaya J.J., Talavera E., Giménez D., Gómez N., Felipe J., Naranjo J.E. (2015). Vulnerable road users detection using V2X communications. Proceedings of the 2015 IEEE 18th International Conference on Intelligent Transportation Systems.

[15] Al-Lawati A., Al-Jahdhami S., Al-Belushi A., Al-Adawi D., Awadalla M., Al-Abri D. (2015). RFID-based system for school children transportation safety enhancement. Proceedings of the 2015 IEEE 8th GCC Conference & Exhibition.

[16] Sewalkar P., Seitz J. (2019). Vehicle-to-pedestrian communication for vulnerable road users: Survey, design considerations, and challenges. Sensors.

[17] Thandavarayan G., Sepulcre M., Gozalvez J. (2019). Analysis of Message Generation Rules for Collective Perception in Connected and Automated Driving. Proceedings of the 2019 IEEE Intelligent Vehicles Symposium (IV).

[18] Garlichs K., Günther H.J., Wolf L.C. (2019). Generation Rules for the Collective Perception Service. Proceedings of the IEEE Vehicular Networking Conference (VNC).

[19] Wu X., Miucic R., Yang S., Al-Stouhi S., Misener J., Bai S., Chan W.h. (2014). Cars talk to phones: A DSRC based vehicle-pedestrian safety system. Proceedings of the 2014 IEEE 80th Vehicular Technology Conference (VTC2014-Fall).

[20] FCC Modernizes 5.9 GHz Band to Improve Wi-Fi and Automotive Safety. https://www.fcc.gov/document/fcc-modernizes-59-ghz-band-improve-wi-fi-and-automotive-safety.

[21] European Telecommunications Standards Institute Intelligent Tranportation Systems (ETSI ITS) (2019). Intelligent Transport Systems (ITS); Vehicular Communications; Basic Set of Applications; Part 2: Specification of Cooperative Awareness Basic Service.

[22] Lee S., Kim D. (2016). An energy efficient vehicle to pedestrian communication method for safety applications. Wirel. Pers. Commun..

[23] Bagheri M., Siekkinen M., Nurminen J.K. (2016). Cloud-based pedestrian road-safety with situation-adaptive energy-efficient communication. IEEE Intell. Transp. Syst. Mag..

[24] Kenney J.B. (2011). Dedicated Short-Range Communications (DSRC) Standards in the United States. Proc. IEEE.

[25] 3rd Generation Partnership Project (3GPP) Technical Specification Group Services and System Aspects; Release 14 Description; Summary of Rel-14 Work Items (Release 14). https://portal.3gpp.org/desktopmodules/Specifications/SpecificationDetails.aspx?specificationId=2934.

[26] 3rd Generation Partnership Project (3GPP) Technical Specification Group Services and System Aspects; Release 15 Description; Summary of Rel-15 Work Items (Release 15). https://portal.3gpp.org/desktopmodules/Specifications/SpecificationDetails.aspx?specificationId=3389.

[27] 3rd Generation Partnership Project (3GPP) Technical Specification Group Services and System Aspects; Enhancement of 3GPP support for V2X scenarios; Stage 1 (Release 15). https://portal.3gpp.org/desktopmodules/Specifications/SpecificationDetails.aspx?specificationId=3180.

[28] Zheng K., Zhang L., Xiang W., Wang W. (2016). Heterogeneous Vehicular Networks.

[29] Valle F., Céspedes S., Hafid A. (2020). Automated Decision System to Exploit Network Diversity for Connected Vehicles. IEEE Trans. Veh. Technol..

[30] Hassan M.I., Vu H.L., Sakurai T. (2011). Performance analysis of the IEEE 802.11 MAC protocol for DSRC safety applications. IEEE Trans. Veh. Technol..

[31] Boban M., d’Orey P.M. (2016). Exploring the practical limits of cooperative awareness in vehicular communications. IEEE Trans. Veh. Technol..

[32] Yáñez A., Céspedes S. (2020). Pedestrians also Have Something to Say: Integration of Connected VRU in Bidirectional Simulations. Proceedings of the 2020 IEEE Vehicular Networking Conference (VNC).

[33] Schuhbäck S., Daßler N., Wischhof L., Köste G. (2019). Towards a Bidirectional Coupling of Pedestrian Dynamics and Mobile Communication Simulation. Proceedings of the 6th International OMNeT++ Community Summit 2019.

[34] Codeca L., Härri J. (2018). Monaco SUMO Traffic (MoST) Scenario: A 3D Mobility Scenario for Cooperative ITS. EPiC Ser. Eng..

[35] Behrisch M., Bieker L., Erdmann J., Knocke M., Krajzewicz D., Wagner P. (2014). Evolution of SUMO’s Simulation Model.

[36] IEEE Computer Society LAN/MAN Standards Committee (2016). IEEE Standard for Information technology–Telecommunications and information exchange between systems Local and metropolitan area networks–Specific requirements Part 11: Wireless LAN Medium Access Control (MAC) and Physical Layer (PHY) Specifications. IEEE Std 802.11-2016 (Revision of IEEE Std 802.11-2012).

[37] Tian B., Hou K.M., Zhou H. (2016). The traffic adaptive data dissemination (TrAD) protocol for both urban and highway scenarios. Sensors.

[38] Sommer C., German R., Dressler F. (2011). Bidirectionally coupled network and road traffic simulation for improved IVC analysis. IEEE Trans. Mob. Comput..

[39] Meinilä J., Kyösti P., Jämsä T., Hentilä L. (2009). WINNER II channel models. Radio Technol. Concepts IMT-Adv..

[40] Ansari K. (2021). Joint use of DSRC and C-V2X for V2X communications in the 5.9 GHz ITS band. IET Intell. Transp. Syst..

[41] Zhao L., Fang J., Hu J., Li Y., Lin L., Shi Y., Li C. (2018). The performance comparison of LTE-V2X and IEEE 802.11 p. Proceedings of the 2018 IEEE 87th Vehicular Technology Conference (VTC Spring).

[42] Ahmed S., Huda M.N., Rajbhandari S., Saha C., Elshaw M., Kanarachos S. (2019). Pedestrian and cyclist detection and intent estimation for autonomous vehicles: A survey. Appl. Sci..

[43] Tahmasbi-Sarvestani A., Mahjoub H.N., Fallah Y.P., Moradi-Pari E., Abuchaar O. (2017). Implementation and evaluation of a cooperative vehicle-to-pedestrian safety application. IEEE Intell. Transp. Syst. Mag..

[44] Fang Z., López A.M. (2019). Intention recognition of pedestrians and cyclists by 2d pose estimation. IEEE Trans. Intell. Transp. Syst..

[45] Torres S., Céspedes S., Bustos-Jiménez J., Serrano M. (2019). IoT solutions for Sustainable Cities: An Online Adaptation for the Driver Intent Inference Algorithm. Proceedings of the 2019 IEEE 5th World Forum on Internet of Things (WF-IoT).

[46] Schulz A.T., Stiefelhagen R. (2015). Pedestrian intention recognition using latent-dynamic conditional random fields. Proceedings of the 2015 IEEE Intelligent Vehicles Symposium (IV).

[47] Quintero R., Parra I., Lorenzo J., Fernández-Llorca D., Sotelo M. (2017). Pedestrian intention recognition by means of a hidden markov model and body language. Proceedings of the 2017 IEEE 20th international conference on intelligent transportation systems (ITSC).

[48] Naik G., Choudhury B., Park J.M. (2019). IEEE 802.11 bd & 5G NR V2X: Evolution of radio access technologies for V2X communications. IEEE Access.

[49] IEEE P802.11-Task Group bd (NGV) Meeting Update. https://www.ieee802.org/11/Reports/tgbd_update.htm.

[50] Chen X., Leng S., Wu F. (2018). Reinforcement Learning Based Safety Message Broadcasting in Vehicular Networks. Proceedings of the 2018 10th International Conference on Wireless Communications and Signal Processing (WCSP).

[51] ETSI (2020). ETSI TS 103 300-2 Intelligent Transport System (ITS); Vulnerable Road Users (VRU) awareness ; Part 2: Functional Architecture and Requirements definition; Release 2.

